# Study protocol: the effect of whole body vibration on acute unilateral unstable lateral ankle sprain- a biphasic randomized controlled trial

**DOI:** 10.1186/1471-2474-14-22

**Published:** 2013-01-14

**Authors:** Sebastian Felix Baumbach, Mariette Fasser, Hans Polzer, Michael Sieb, Markus Regauer, Wolf Mutschler, Matthias Schieker, Michael Blauth

**Affiliations:** 1 Department of Surgery, Campus Innenstadt, Ludwig-Maximilians-University, Munich, Germany; 2 Department of Trauma Surgery, Medical University of Innsbruck, Innsbruck, Austria

**Keywords:** Whole body vibration, Ankle sprain, Rehabilitation, Functional treatment

## Abstract

**Background:**

Ankle sprains often result in ankle instability, which is most likely caused by damage to passive structures and neuromuscular impairment. Whole body vibration (WBV) is a neuromuscular training method improving those impaired neurologic parameters. The aim of this study is to compare the current gold standard functional treatment to functional treatment plus WBV in patients with acute unilateral unstable inversion ankle sprains.

**Methods/Design:**

60 patients, aged 18–40 years, presenting with an isolated, unilateral, acute unstable inversion ankle sprain will be included in this bicentric, biphasic, randomized controlled trial. Samples will be randomized by envelope drawing. All patients will be allowed early mobilization and pain-dependent weight bearing, limited functional immobilization by orthosis, PRICE, NSARDs as well as home and supervised physiotherapy. Supervised physical therapy will take place twice a week, for 30 minutes for a period of 6 weeks, following a standardized intervention protocol. During supervised physical therapy, the intervention group will perform exercises similar to those of the control group, on a side-alternating sinusoidal vibration platform. Two time-dependent primary outcome parameters will be assessed: short-term outcome after six weeks will be postural control quantified by the sway index; mid-term outcome after one year will be assessed by subjective instability, defined by the presence of giving-way attacks. Secondary outcome parameters include: return to pre-injury level of activities, residual pain, recurrence, objective instability, energy/coordination, Foot and Ankle Disability Index and EQ 5D.

**Discussion:**

This is the first trial investigating the effects of WBV in patients with acute soft tissue injury. Inversion ankle sprains often result in ankle instability, which is most likely due to damage of neurological structures. Due to its unique, frequency dependent, influence on various neuromuscular parameters, WBV is a promising treatment method for patients with acute unstable inversion ankle sprains.

**Trial registration:**

NCT01702597

## Background

Lateral ankle sprains are one of the most common musculoskeletal injuries [1,2]. They mostly result from an internal rotation and adduction of the plantarflexed foot with subsequent damage to the lateral capsulo-ligamentous complex. About 65% of all lateral ankle sprains are isolated anterior tibio-fibular ligament (ATFL) injuries, while 20% are combined ATFL and calcano-fibular ligament injuries [3]. 85% of all ankle injuries are ankle sprains and 85% of those are inversion sprains [4], with about one ankle sprain occurring per 10.000 people every day [5]. Although usually considered an innocuous injury, three-year full recovery rates range from 36% to 85% [6].

Ankle instability is most likely caused by damage to passive structures and neuromuscular impairment [7]. Damage to passive structures, i.e. capsular structure and ligaments, results in objective (anterior drawer, talar tilt) and subjective instability (giving-way) [3]. Neurological impairments include muscle fatigue [7-9], reduced dynamic balance [10-12] and impaired postural control [13-15]. Although not all patients with functional (FAI) or chronic ankle instability (CAI) present with objective instability, they are all thought to have neuromuscular impairments [2,16,17]. This results in lower physical activity levels [18], diminished quality of life [19], and a possible increase in the risk of osteoarthritis [20,21].

Any treatment plan should therefore address both damaged passive structures and neuromuscular impairment. Generally, three treatment regimens are available: functional treatment (early mobilization and bracing), cast immobilization for 2–6 weeks, or operative treatment [22,23]. A Cochrane review from 2007 comparing conservative and surgical treatments found similar outcomes [3]. Therefore, functional treatment has to be considered the current gold standard. Functional treatment should allow healing of damaged passive structures and compensate for neuromuscular impairments, i.e. improve muscle strength and proprioception. Physical therapy therefore includes stretching, body-weight exercises, training apparatus and devices (for example wobble board training). Supervised rehabilitation has proven superior to conventional treatment [24,25].

A neuromuscular training method gaining increased attention is whole body vibration (WBV). WBV involves synchronous or side-alternating sinusoidal vibrations, which are transmitted to the body via platforms. It is believed to evoke muscle contractions via stretch reflexes in the muscle spindle system [26-28]. A growing body of evidence indicates improvements of various neuromuscular parameters following WBV, such as power, strength, movement velocity, range of motion and balance [29-38]. Reports to the contrary [39,40] may be due to heterogeneous patient populations and varying intervention protocols, which in turn can be due to limited knowledge of the dose-effect relationship, i.e. vibration frequency and amplitude.

In general, frequencies <10Hz are believed to loosen muscle and tissue. Frequencies >10Hz and <20Hz still allow active contraction and relaxation of muscle fibers and are used for coordination exercises. Frequencies >20Hz result in isotonic muscle contraction [41]. Vibration amplitude seems to be positively correlated to muscle activity [42-44]. Although WBV has numerous contraindications, as listed in Table 1, the only adverse side effects reported are temporary hyposensitivity of the foot soles [45].

**Table 1:** Inclusion- and exclusion criteria

**Inclusion criteria**	**Exclusion criteria**
Age: 18 to 40 years	Pregnancy
Acute, unilateral, unstable, inversion ankle sprain (Grade II, III)	Conditions affecting the neuromuscular or musculoskeletal system
Signed informed consent	Previous surgical interventions to the foot, ankle, knee or hip; known FAI, CAI
Patient can read and understand German	Conditions possibly affecting balance
	Cardiovascular disease including thrombosis
	Respiratory diseases
	Abdominal diseases (including gallstones)
	Urological diseases (including kidney and bladder stones)
	Gynaecological diseases and + intrauterine devices
	Neurological diseases including epilepsy within the last 2 years
	Acute injuries to the head
	Patient is not available for follow-up visits
	Patient unable to give informed consent
	Patient suspected to be non-compliant

Functional classification system for lateral ankle sprains: Grade II: hematoma/swelling/pain on palpation and positive anterior drawer test (complete tear of the ATFL, incomplete tear of the CFL); Grade III: hematoma/swelling/pain on palpation and positive anterior drawer test and positive talar tilt test (complete tear of the ATFL and CFL); adapted from [46-48]; FAI: Functional ankle instability; CAI: Chronic ankle instability.

With WBV improving range of motion (ROM), power and balance - parameters known to be affected in patients with ankle instability - it might be beneficial to include it into the current functional treatment regime. To our knowledge only one study investigated the effects of WBV in patients with ankle instability. Cloak at al. [49] conducted a randomized controlled trial (RCT) on functional ankle instability in dancers (n=38), comparing WBV to a control group (regular dance training). The WBV group yielded significantly better results for balance (STAR extrusion balance test) as well as a significantly reduced Center of Pressure values.

### Aim of study

The aim of this bicentric, biphasic, randomized, controlled study is to compare current gold standard functional treatment to functional treatment plus WBV in patients with acute unilateral unstable inversion ankle sprains over a period of 12 months.

### Hypotheses

1. Short term results (after 6 weeks): H_0_ = Functional treatment in combination with WBV therapy in patients with acute unilateral unstable inversion ankle sprains does not improve the Sway-Index compared to functional treatment alone.

2. Mid-term results (after 12 months): H_0_ = Functional treatment in combination with WBV therapy in patients with acute unilateral unstable inversion ankle sprains does not result in a reduction of the recurrence rate compared to functional treatment alone.

## Methods/Design

### Study design and protocol

**Figure 1:**
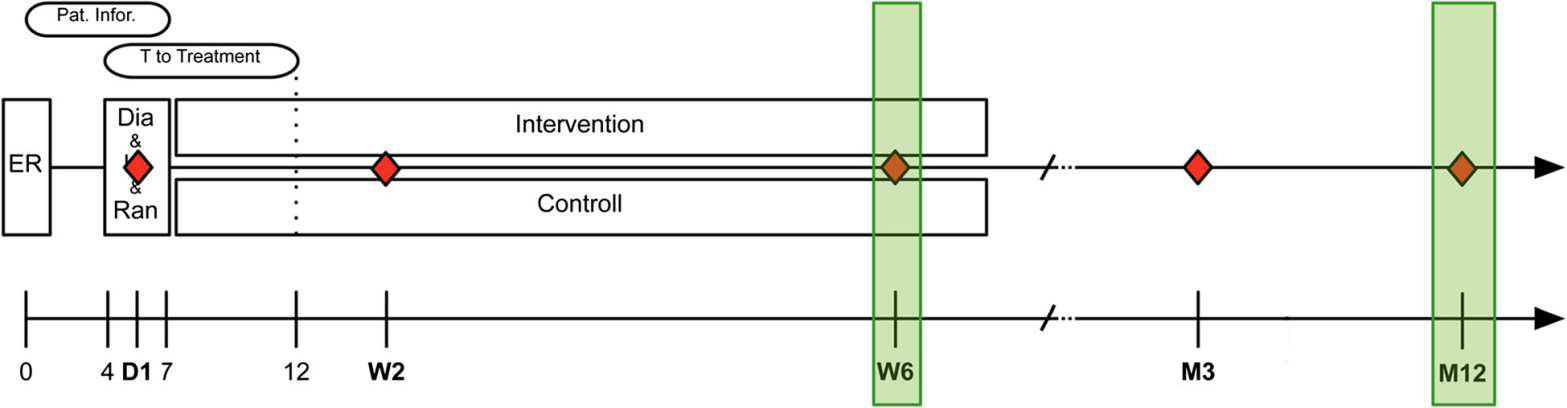
**Schematic illustration of the study protocol.** ER = Emergency Department; Dia & Inc & Ran = Diagnosis, inclusion and randomization of patient; Pat. inform = Patient information; T to Treatment = maximum time interval between patient inclusion and first treatment intervention; D = day; W = week; M = month; Red diamond = Patient visits; Green segments = short-term/mid-term analysis.

The study design is a bicentric, biphasic, randomized, controlled trial, following the CONSORT statement guidelines [50]. 60 patients will be randomized into an intervention (supervised functional treatment and WBV) or control group (supervised functional treatment). Intervention will take place twice a week at a rate of 30 minutes per session, for 6 weeks. Figure 1 schematically illustrates the study protocol.

The study design is in accordance with the recommendations of the Declaration of Helsinki, and was approved by the Ethical Committee of the Medical University of Munich (#315-12) and the Medical University of Innsbruck (#UN4833). The study is registered as a randomized controlled trial (NCT01702597).

### Study centers, population, screening and randomization

The study will be conducted at the Department of Surgery, Medical University of Munich (LMU), Germany, and the Department of Trauma Surgery, Medical University of Innsbruck, Austria. Patients will be screened within the regular emergency unit setting using a standardized algorithm for ankle sprains [51]. Patients presenting with an isolated, unilateral, acute unstable ankle sprain grade II or III (as defined in Table 1), aged 18–40 years, will be informed about the study and potential risks before signing the informed consent. In case initial classification is not possible (i.e. patients do not tolerate medical examinations), patients will be invited for a delayed physical examination four to seven days following trauma. Inclusion and exclusion criteria are listed in Table 1. Within seven days, participating patients must be included and randomized by envelope drawing.

### Intervention

All patients will be allowed early mobilization and pain-dependent weight bearing, functional immobilization by orthosis, PRICE (protection, rest, ice, compression, elevation), NSAIDs as well as home and supervised physiotherapy. Patients will receive a standardized handout for home training. Table 2 outlines the standardized supervised intervention protocol for both groups, taking into consideration the different soft tissue healing phases. The functional treatment protocol was developed based on treatment guidelines published by the Royal Dutch Society of Physical Therapy [52] and is similar to previous treatment protocols [53-56]. Patients will receive supervised physiotherapy twice a week for 6 weeks [57-59] with each session lasting 30 minutes. Patients will be asked to do home exercises according to the handout every second day.

**Table 2:** Detailed treatment protocol

**Lvl**	**Phases**	**Time**	**Symptoms**	**Treatment Goals**	**Physiotherapy:**	**Physiotherapy:**
					**CONTROL GROUP**	**INTERVENTION GROUP**^**1**^
1	Inflam.	0 - 3d	▪ Pain at rest	▪ Reduction of pain and swelling	▪ PRICE	
			▪ Swelling and hematoma	▪ Improvement of perfusion	▪ NSARDs	
			▪ Pain during weight bearing	▪ Partial weight bearing	▪ Pain-dependent weight bearing +/- crutches	
					▪ Pain-dependent mobilization of the foot	
					▪ No tape or brace (due to swelling)	
2	Prolif.	4 - 10d	▪ Foot can actively be put into neutral position	▪ Restoring function	▪ NSARDs	Frequency 10Hz / 16 Hz
			▪ Reduction of swelling	▪ Restoring full weight bearing	▪ Pain-dependent weight bearing +/- crutches	Amplitude: s. below
			▪ Partial weight bearing without complete heel-to-toe movement		▪ Arch of foot / leg axis	Duration: 5min
			▪ Possible fear of movement		▪ Training of symmetrical gait and regular foot strike	Exercises, vibration:
					▪ Exercises to improve ROM, active stabilization, coordination and regular walking pattern	▪ Gymnastic ball, feet parallel to mark 1 or 2, patient rolls forth and back, in order to Flex/Ext. in the upper ankle joint
					▪ Brace	▪ Gymnastic ball, injured foot placed transverse on the WBV platform (ankle in-between mark 0 and 1), patient rolls forth and back, in order to flex/extent in the upper ankle joint
						Exercises, general:
						▪ Pain dependent weight bearing +/- Crutches
						▪ Walking motion training
3	Early Remod.	11 - 21d	▪ Residual hematoma	▪ Improving muscular strength, and active/ functional ankle stability, and ROM	▪ Information on preventive measures (Brace)	Frequency: >10Hz, 18-24Hz
			▪ Normal heal-to-toe movement	▪ Training regular walking pattern and climbing stairs	▪ Exercises to improve balance, ROM, muscle strength, walking pattern, running and climbing the stairs	Amplitude: 1-2,5mm
			▪ Pain and fear of movement under load		▪ Dynamic stability: stepwise increase of training intensity; switching from static to dynamic exercises	Duration: 3 Sets a 3 Min with 2 Min break each
					▪ Guidance for home training	Exercises, vibration:
						1) Dynamic squatting (warm-up)
						2) Dynamic squatting (increasing depth)
						3) Two leg stance with slightly bend knees, slow weight transfer (right ←→ left)
						4) One-leg squatting, transverse to WBV plate +/- support of the non-injured leg
						Exercises, general:
						▪ Guidance for home training
4	Late Remod.	3 - 6wk	▪ No hematoma	▪ Improvement of resistance during walking, running, climbing stairs	▪ Exercises to improve coordination (skipping, jumping, …)	Frequency: >10Hz, 18-24Hz
			▪ Dorsal flexion possible	▪ Improvement of work/sports specific tasks	▪ Stepwise load increase and switching from static to dynamic / from simple to complex / from cyclic to non-cyclic exercises	Amplitude: 2-3mm
			▪ No more pain or fear of movement during sports		▪ Guidance for home training	Duration: 3 Sets a 3 Min with 2 Min break each
						Exercises, vibration:
						1) Dynamic squatting (warm-up)
						2) Side-skipping with flexed knees
						3) Calf raises
						4) Vibration on a tilted surface, elevated leg = uninjured leg
						5) Static squats (45° / 90°); Frequency 18+, Amplitude 2+
						Exercises, general:
						▪ Guidance for home training

Lvl: Level; Phase: Healing phases; d: days; Remod.: Remodeling; Inflam.: Inflammatory phase; Prolif.: Proliferation phase; wk: week; PRICE: Protection, Rest, Ice, Compression, Elevation; NSARDs: Non-steroidal antirheumatic drugs; ROM: Range of motion; min: Minutes; Hz: Herz; mm: Millimeter; WBV: Whole body vibration; Min: Minute.

The intervention group will use a sinusoidal side alternating vibration system (Galileo® Med M Plus, Novotec, Pforzheim, Germany) with a peak-to-peak displacement range from 0–9 with 5–30 Hz acceleration. They will perform similar exercises as the control group on the vibration platform and the overall session duration will be the same. The detailed WBV intervention protocol is presented in the Additional file 1.

Physiotherapists who will receive specific training for the WBV prior to this study will supervise all training sessions.

### Outcome parameters

The in Table 3 outlined outcome parameters are the most frequently used in ankle sprain literature [3,22,25]. Neuromuscular impairment will be assessed using a Leonardo Mechanograph GRFP (16 bit, 800 Hz; NOVOTEC Medical GmbH, Germany) and are marked with a * in Table 3. Each test will be performed following the manufacturer’s instructions, with the patients not having participated in sports 24h prior to data acquisition. Tests will be conducted as soon as tolerated by patient.

**Table 3:** Complete list of outcome parameters


**Primary Outcome parameter (1)**	
(short term; 6 weeks)	
Postural control: Balance Test (Sway Index)*	[numeric, scale]
	
**Primary Outcome parameter (2)**	
(mid-term; 1 year)	
Subjective Instability (Giving-way)	[dichotomous variable]
	
**Secondary Outcome parameters**	
Return to pre-injury level of activity (work, sports)	[dichotomous variable]
Residual pain:	
Pain at rest	[dichotomous variable; VAS]
Pain on weight-bearing	[dichotomous variable; VAS]
Pain during sports	[dichotomous variable; VAS]
Subjective Instability (Giving-way)	[dichotomous variable]
Recurrence	[dichotomous variable]
Objective instability:	
Anterior drawer	[dichotomous variable]
Talar tilt	[dichotomous variable]
Ankle ROM	[numeric, scale]
Postural control: Balance Test (Sway index)*	[numeric, scale]
Energy/coordination:	
Multiple one leg hopping*	[numeric, scale]
Single two leg jump*	[numeric, scale]
Complications	[text]
Scores:	
Foot and Ankle Disability Index [60]	[numeric, scale]
EQ 5D 5L	[numeric, scale]

### Sample size estimation and statistics

With this study being the first using WBV with acutely injured patients, data to conduct sample size estimation is lacking. Therefore sample size was chosen according to previous studies on WBV or ankle instability [32,49,61,62].

Statistical methods used will include descriptive statistics, Student’s *t*-test and Fisher’s exact test.

### Adverse events

The only adverse side effects of WBV reported in literature were temporary hyposensitivity of the foot soles [45]. Possible adverse events related to the interventions include pain, fall from the WBV device/wobble board, and delayed mobilization or healing. Unexpected events related to data acquisition might be patients’ inability to perform certain load-dependent examinations. Severe adverse events are only expected following a fall from the training device.

## Discussion

The present study protocol on the effect of WBV in patients with acute, unilateral, unstable inversion ankle sprains is the first study to apply WBV in patients with acute soft tissue injuries. Due to its influence on various neuromuscular parameters, which are known to be impaired in patients with ankle instability, WBV provides a novel, functional treatment approach for this problem.

Several limitations of the present protocol must be discussed. First of all, the authors have decided to use a functional classification system. With ankle instability being the most important parameter for further treatment decision, an established functional classification system was chosen. Ankles are classified into stable or unstable based on clinical presentation and examination (swelling, anterior drawer test and talar tilt test) [46-48]. In case initial examination is not tolerated, delayed physical examination has been shown to be equal to arthrography (specificity/sensitivity: 85%/96%) [63-65]. Its feasibility in daily practice and high sensitivity makes it a suitable classification system for patients with acute inversion ankle sprains.

Second, as discussed in the introduction, functional treatment seems to be the current gold standard treatment approach. Still, immobilization, as well as type and duration of physiotherapy are a matter of discussion. Kerkhoffs et al. [66] conducted a systematic review on the effectiveness of various braces/bracing methods for acute ankle sprains and found lace-up supports to be the most effective, which seems supported by a Cochrane Review [3]. A recent single-blinded RCT by Lamb et al. [67] found a short period of immobilization in an Aircast brace to result in faster recovery than double-layered tubular compression bandage. This is in line with a review by Kemle et al. [23] who found evidence pointing towards the superiority of ankle braces. Consequently, initial immobilization will be realized with an ankle brace. Moreover, the type and duration of functional rehabilitation remains unclear. Our supervised rehabilitation protocol is based on the guidelines of the Royal Dutch Society of Physical Therapy [52], which is comparable to other published treatment protocols [53-57,68]. Although the optimal intensity of physical rehabilitation remains unclear, 45 minutes, twice to three times a week over a period of 6–8 weeks seem to be beneficial [10,57-59,69]. Due to administrative issues, we decided on supervised rehabilitation for 30 minutes, twice weekly for 6 weeks, with additional home exercises.

A third possible limitation are the chosen outcome parameters. Whereas power, assessed by a single leg vertical jump, has been proven significantly different between patients with recurrent ankle sprains and healthy controls [14,70-72], there is no consensus in literature as to which method best assesses balance. A commonly used method is center of pressure (COP) [73-76]. Whereas COP assessed during single leg stance with eyes open was ineffective in identifying patients with ankle instability [73,74], this could be achieved with single leg stance with eyes closed [75,76]. Furthermore, COP provides an objectively verifiable parameter. Other commonly used outcome parameters such as return to work or return to sports are parameters not necessarily coinciding with the recovery of the injured ankle. They highly depend on the patient’s attitude attitude, schedules and type of job/sport.

## Abbreviations

WBV: Whole Body Vibration; PRICE: Protection, Rest, Ice, Compression, Elevation; NSARDs: Non-Steroidal Antirheumatic Drugs; %: Percent; ATFL: Anterior Tibio-Fibular Ligament; FAI: Functional Ankle Instability; CAI: Chronic Ankle Instability; Hz: Herz; ROM: Range Of Motion; RCT: Randomized Controlled Trial; n: Number; H_0_: Hypothesis; h: Hour; COP: Center Of Pressure.

## Competing interest

The authors declare that they have no competing interests.

## Authors’ contributions

SFB designed the study, was responsible for ethical approval of the LMU, Munich and prepared the manuscript. MW substantially contributed to the conception of the study protocol, was responsible for the ethical approval in Innsbruck and assisted in drafting the manuscript. HP had substantial input in the design of the study and revised the manuscript. MS (Dr. Sieb) substantially contributed to the conception of the study, revised the ethics proposal for Innsbruck and revised the manuscript. MR contributed to the design of the study, revised the ethics proposal for Munich and the manuscript. WM contributed to the design of the study, revised the ethics proposal for Munich and the manuscript. MS (Prof. Schieker) supervised the study protocol preparation and revised the manuscript. MB substantially contributed to the design of the study, was responsible for the ethics proposal for Innsbruck and revised the manuscript. All authors read and approved the final manuscript.

## Pre-publication history

The pre-publication history for this paper can be accessed here:

http://www.biomedcentral.com/1471-2474/14/22/prepub

## Supplementary Material

Additional file 1 Detailed WBV treatment protocol. Click here for file

## References

[B1] McCulloch PG Holden P Robson DJ Rowley DI Norris SH The value of mobilisation and non-steroidal anti-inflammatory analgesia in the management of inversion injuries of the ankle Br J Clin Pract 1985 39 69 72 3872674

[B2] Hertel J Functional anatomy, pathomechanics, and pathophysiology of lateral ankle instability J Athl Train 2002 37 364 375 12937557 PMC164367

[B3] Kerkhoffs GMMJ Struijs PAA Marti RK Assendelft WJJ Blankevoort L van Dijk CN Different functional treatment strategies for acute lateral ankle ligament injuries in adults Cochr Database Syst Rev (Online) 2007 CD002938 10.1002/14651858.CD002938 12137665

[B4] Sheth P Yu B Laskowski ER An KN Ankle disk training influences reaction times of selected muscles in a simulated ankle sprain Am J Sports Med 1997 25 538 543 9240989 10.1177/036354659702500418

[B5] Kannus P Renström P Treatment for acute tears of the lateral ligaments of the ankle. Operation, cast, or early controlled mobilization J Bone Joint Surg Am Vol 1991 73 305 312 1993726

[B6] van Rijn RM van Os AG Bernsen RMD Luijsterburg PA Koes BW Bierma-Zeinstra SMA What is the clinical course of acute ankle sprains? a systematic literature review Am J Med 2008 121 324 331 e326 18374692 10.1016/j.amjmed.2007.11.018

[B7] Palmieri-Smith RM Hopkins JT Brown TN Peroneal activation deficits in persons with functional ankle instability Am J Sports Med 2009 37 982 988 19270189 10.1177/0363546508330147

[B8] Gribble PA Hertel J Effect of lower-extremity muscle fatigue on postural control Arch Phys Med Rehabil 2004 85 589 592 15083434 10.1016/j.apmr.2003.06.031

[B9] Mitchell A Dyson R Hale T Abraham C Biomechanics of ankle instability. Part 1: reaction time to simulated ankle sprain Med Sci Sports Exerc 2008 40 1515 1521 18705024 10.1249/mss.0b013e31817356b6

[B10] Mckeon PO Ingersoll CD Kerrigan DC Saliba E Bennett BC Hertel J Balance training improves function and postural control in those with chronic ankle instability Med Sci Sports Exerc 2008 40 1810 1819 18799992 10.1249/MSS.0b013e31817e0f92

[B11] Hardy L Huxel K Brucker J Nesser T Prophylactic ankle braces and star excursion balance measures in healthy volunteers J Athl Train 2008 43 347 351 18668181 10.4085/1062-6050-43.4.347 PMC2474828

[B12] Eechaute C Vaes P Duquet W The dynamic postural control is impaired in patients with chronic ankle instability: reliability and validity of the multiple hop test Clin J Sport Med Offic J Can Acad Sport Med 2009 19 107 114 10.1097/JSM.0b013e3181948ae8 19451764

[B13] Sesma AR Mattacola CG Uhl TL Nitz AJ Mckeon PO Effect of foot orthotics on single- and double-limb dynamic balance tasks in patients with chronic ankle instability Foot Ankle Spec 2008 1 330 337 19825736 10.1177/1938640008327516

[B14] Ross SE Guskiewicz KM Gross MT Yu B Balance measures for discriminating between functionally unstable and stable ankles Med Sci Sports Exerc 2009 41 399 407 19127184 10.1249/MSS.0b013e3181872d89

[B15] Sefton JM Hicks-Little CA Hubbard TJ Clemens MG Yengo CM Koceja DM Cordova ML Sensorimotor function as a predictor of chronic ankle instability Clin Biomech (Bristol, Avon) 2009 24 451 458 10.1016/j.clinbiomech.2009.03.003 19346037

[B16] Tropp H Odenrick P Gillquist J Stabilometry recordings in functional and mechanical instability of the ankle joint Int J Sports Med 1985 6 180 182 4030196 10.1055/s-2008-1025836

[B17] Becker HP Rosenbaum D Chronic recurrent ligament instability on the lateral ankle Der Orthopäde 1999 28 483 492 10431303 10.1007/s001320050375

[B18] Verhagen RA de Keizer G van Dijk CN Long-term follow-up of inversion trauma of the ankle Arch Orthop Trauma Surg 1995 114 92 96 7734241 10.1007/BF00422833

[B19] Anandacoomarasamy A Barnsley L Long term outcomes of inversion ankle injuries Br J Sports Med 2005 39 e14 discussion e14 15728682 10.1136/bjsm.2004.011676 PMC1725165

[B20] Krips R Brandsson S Swensson C van Dijk CN Karlsson J Anatomical reconstruction and evans tenodesis of the lateral ligaments of the ankle. Clinical and radiological findings after follow-up for 15 to 30 years J Bone Joint Surg Br Vol 2002 84 232 236 10.1302/0301-620x.84b2.12143 11924653

[B21] Valderrabano V Hintermann B Horisberger M Fung TS Ligamentous posttraumatic ankle osteoarthritis Am J Sports Med 2006 34 612 620 16303875 10.1177/0363546505281813

[B22] Pijnenburg AC Van Dijk CN Bossuyt PM Marti RK Treatment of ruptures of the lateral ankle ligaments: a meta-analysis J Bone Joint Surg Am Vol 2000 82 761 773 10.2106/00004623-200006000-00002 10859095

[B23] Kemler E van de Port I Backx F van Dijk CN A systematic review on the treatment of acute ankle sprain: brace versus other functional treatment types Sports Med (Auckland, NZ) 2011 41 185 197 10.2165/11584370-000000000-00000 21395362

[B24] van Os AG Bierma-Zeinstra SMA Verhagen AP de Bie RA Luijsterburg PAJ Koes BW Comparison of conventional treatment and supervised rehabilitation for treatment of acute lateral ankle sprains: a systematic review of the literature J Orthop Sports Phys Ther 2005 35 95 105 15773567 10.2519/jospt.2005.35.2.95

[B25] van Rijn RM van Ochten J Luijsterburg PAJ van Middelkoop M Koes BW Bierma-Zeinstra SMA Effectiveness of additional supervised exercises compared with conventional treatment alone in patients with acute lateral ankle sprains: systematic review BMJ 2010 341 c5688 20978065 10.1136/bmj.c5688 PMC2965125

[B26] Rittweger J Ehrig J Just K Kirsch MMA Felsenberg D Oxygen uptake in whole-body vibration exercise: influence of vibration frequency, amplitude, and external load Int J Sports Med 2002 23 428 432 12215962 10.1055/s-2002-33739

[B27] Rittweger J Mutschelknauss M Felsenberg D Acute changes in neuromuscular excitability after exhaustive whole body vibration exercise as compared to exhaustion by squatting exercise Clin Physiol Funct Imaging 2003 23 81 86 12641601 10.1046/j.1475-097x.2003.00473.x

[B28] Kvorning T Bagger M Caserotti P Madsen K Effects of vibration and resistance training on neuromuscular and hormonal measures Eur J Appl Physiol 2006 96 615 625 16482475 10.1007/s00421-006-0139-3

[B29] Cheung W-H Mok H-W Qin L Sze P-C Lee K-M Leung K-S High-frequency whole-body vibration improves balancing ability in elderly women Arch Phys Med Rehabil 2007 88 852 857 17601464 10.1016/j.apmr.2007.03.028

[B30] Torvinen S Kannus P Sievänen H Järvinen TAH Pasanen M Kontulainen S Nenonen A Järvinen TLN Paakkala T Järvinen M Vuori I Effect of 8-month vertical whole body vibration on bone, muscle performance, and body balance: a randomized controlled study J Bone Miner Res 2003 18 876 884 12733727 10.1359/jbmr.2003.18.5.876

[B31] Delecluse C Roelants M Verschueren S Strength increase after whole-body vibration compared with resistance training Med Sci Sports Exerc 2003 35 1033 1041 12783053 10.1249/01.MSS.0000069752.96438.B0

[B32] Cochrane DJ Stannard SR Acute whole body vibration training increases vertical jump and flexibility performance in elite female field hockey players Br J Sports Med 2005 39 860 865 16244199 10.1136/bjsm.2005.019950 PMC1725065

[B33] Verschueren SMP Roelants M Delecluse C Swinnen S Vanderschueren D Boonen S Effect of 6-month whole body vibration training on hip density, muscle strength, and postural control in postmenopausal women: a randomized controlled pilot study J Bone Miner Res 2004 19 352 359 15040822 10.1359/JBMR.0301245

[B34] Bogaerts A Verschueren S Delecluse C Claessens AL Boonen S Effects of whole body vibration training on postural control in older individuals: a 1 year randomized controlled trial Gait Posture 2007 26 309 316 17074485 10.1016/j.gaitpost.2006.09.078

[B35] Rees SS Murphy AJ Watsford ML Effects of whole body vibration on postural steadiness in an older population J Sci Med Sport 2009 12 440 444 18550436 10.1016/j.jsams.2008.02.002

[B36] Cardinale M Bosco C The use of vibration as an exercise intervention Exerc Sport Sci Rev 2003 31 3 7 12562163 10.1097/00003677-200301000-00002

[B37] Armstrong WJ Nestle HN Grinnell DC Cole LD Van Gilder EL Warren GS Capizzi EA The acute effect of whole-body vibration on the hoffmann reflex J Strength Condit Res Nat Strength Condit Assoc 2008 22 471 476 10.1519/JSC.0b013e3181660605 18550962

[B38] Lau RW Liao L-R Yu F Teo T Chung RC Pang MY The effects of whole body vibration therapy on bone mineral density and leg muscle strength in older adults: a systematic review and meta-analysis Clin Rehabil 2011 25 975 988 21849376 10.1177/0269215511405078

[B39] Torvinen S Sievänen H Järvinen TA Pasanen M Kontulainen S Kannus P Effect of 4-min vertical whole body vibration on muscle performance and body balance: a randomized cross-over study Int J Sports Med 2002 23 374 379 12165890 10.1055/s-2002-33148

[B40] Cochrane DJ Legg SJ Hooker MJ The short-term effect of whole-body vibration training on vertical jump, sprint, and agility performance J Strength Condit Res Nat Strength Condit Assoc 2004 18 828 832 10.1519/14213.1 15574090

[B41] Rittweger J Just K Kautzsch K Reeg P Felsenberg D Treatment of chronic lower back pain with lumbar extension and whole-body vibration exercise: a randomized controlled trial Spine 2002 27 1829 1834 12221343 10.1097/00007632-200209010-00003

[B42] Cardinale M Lim J Electromyography activity of vastus lateralis muscle during whole-body vibrations of different frequencies J Strength Condit Res Nat Strength Condit Assoc 2003 17 621 624 10.1519/1533-4287(2003)017<0621:eaovlm>2.0.co;2 12930196

[B43] Delecluse C Roelants M Diels R Koninckx E Verschueren S Effects of whole body vibration training on muscle strength and sprint performance in sprint-trained athletes Int J Sports Med 2005 26 662 668 16158372 10.1055/s-2004-830381

[B44] Pollock RD Woledge RC Mills KR Martin FC Newham DJ Muscle activity and acceleration during whole body vibration: effect of frequency and amplitude Clin Biomech (Bristol, Avon) 2010 25 840 846 10.1016/j.clinbiomech.2010.05.004 20541297

[B45] Pollock RD Provan S Martin FC Newham DJ The effects of whole body vibration on balance, joint position sense and cutaneous sensation Eur J Appl Physiol 2011 111 3069 3077 21455611 10.1007/s00421-011-1943-y

[B46] Ferran NA Maffulli N Epidemiology of sprains of the lateral ankle ligament complex Foot Ankle Clin 2006 11 659 662 16971255 10.1016/j.fcl.2006.07.002

[B47] Clanton TO Porter DA Primary care of foot and ankle injuries in the athlete Clin Sports Med 1997 16 435 466 9209820 10.1016/s0278-5919(05)70034-x

[B48] Chorley JN Hergenroeder AC Management of ankle sprains Pediatr Ann 1997 26 56 64 9007971 10.3928/0090-4481-19970101-11

[B49] Cloak R Nevill AM Clarke F Day S Wyon MA Vibration training improves balance in unstable ankles Int J Sports Med 2010 31 894 900 21072738 10.1055/s-0030-1265151

[B50] Schulz KF Altman DG Moher D Group C CONSORT 2010 statement: updated guidelines for reporting parallel group randomised trials BMJ 2010 340 c332 20332509 10.1136/bmj.c332 PMC2844940

[B51] Polzer H Kanz KG Prall WC Haasters F Ockert B Mutschler W Grote S Diagnosis and treatment of acute ankle injuries: development of an evidence-based algorithm Orthop Rev 2012 4 e5 10.4081/or.2012.e5 PMC3348693 22577506

[B52] Wees PV Lenssen AF Feijts YAEJ Bloo H van Morsel SR Ouderland R Opraus KWF Rondhuis G Simons A Swinkels RAHM KNGF guideline for physical therapy in patients with acute ankle sprain - practice guidelines Suppl Dutch J Phys Ther 2006 116 1 30

[B53] Oostendorp R Functionele instabiliteit na het inversietrauma van enkel en voet: een effectonderzoek zleisterbandage versus pleisterbandage zecombineerd met fysiotherapie. [Functional instability after ankle sprains; a trial of taping versus taping and exercise] Geneeskd Sport 1987 20 45 55

[B54] Karlsson J Eriksson BI Swärd L Early functional treatment for acute ligament injuries of the ankle joint Scand J Med Sci Sports 1996 6 341 345 9046544 10.1111/j.1600-0838.1996.tb00104.x

[B55] Bassett SF Prapavessis H Home-based physical therapy intervention with adherence-enhancing strategies versus clinic-based management for patients with ankle sprains Phys Ther 2007 87 1132 1143 17609331 10.2522/ptj.20060260

[B56] van Rijn RM van Os AG Kleinrensink G-J Bernsen RM Verhaar JA Koes BW Bierma-Zeinstra SM Supervised exercises for adults with acute lateral ankle sprain: a randomised controlled trial Br J Gen Pract 2007 57 793 800 17925136 PMC2151811

[B57] Holme E Magnusson SP Becher K Bieler T Aagaard P Kjaer M The effect of supervised rehabilitation on strength, postural sway, position sense and re-injury risk after acute ankle ligament sprain Scand J Med Sci Sports 1999 9 104 109 10220845 10.1111/j.1600-0838.1999.tb00217.x

[B58] Sekir U Yildiz Y Hazneci B Ors F Aydin T Effect of isokinetic training on strength, functionality and proprioception in athletes with functional ankle instability Knee Surg Sports Traumatol Arthrosc 2007 15 654 664 16770637 10.1007/s00167-006-0108-8

[B59] Mckeon PO Hertel J Systematic review of postural control and lateral ankle instability, part II: is balance training clinically effective? J Athl Train 2008 43 305 315 18523567 10.4085/1062-6050-43.3.305 PMC2386424

[B60] Martin RL Burdett RG Irrgang JJ Development of the foot and ankle disability index (FADI) [abstract] J Orthop Sports Phys Ther 1999 29 A32 A33

[B61] de Ruiter CJ van der Linden RM van der Zijden MJA Hollander AP de Haan A Short-term effects of whole-body vibration on maximal voluntary isometric knee extensor force and rate of force rise Eur J Appl Physiol 2003 88 472 475 12527980 10.1007/s00421-002-0723-0

[B62] Jacobs PL Burns P Acute enhancement of lower-extremity dynamic strength and flexibility with whole-body vibration J Strength Condit Res Nat Strength Condit Assoc 2009 23 51 57 10.1519/JSC.0b013e3181839f19 18824930

[B63] van Dijk CN On diagnostic strategies in patients with severe ankle sprain (thesis) 1994 Amsterdam, The Netherlands: Univ of Amsterdam

[B64] van Dijk CN Lim LS Bossuyt PM Marti RK Physical examination is sufficient for the diagnosis of sprained ankles J Bone Joint Surg Br Vol 1996 78 958 962 10.1302/0301-620x78b6.1283 8951015

[B65] Klenerman L The management of sprained ankle J Bone Joint Surg Br Vol 1998 80 11 12 10.1302/0301-620x.80b1.8037 9460944

[B66] Kerkhoffs GMMJ Struijs PAA Marti RK Blankevoort L Assendelft WJJ van Dijk CN Functional treatments for acute ruptures of the lateral ankle ligament: a systematic review Acta Orthop Scand 2003 74 69 77 12635797 10.1080/00016470310013699

[B67] Lamb SE Marsh JL Hutton JL Nakash R Cooke MW Group CASTC Mechanical supports for acute, severe ankle sprain: a pragmatic, multicentre, randomised controlled trial Lancet 2009 373 575 581 19217992 10.1016/S0140-6736(09)60206-3

[B68] Reinhard C Tiedemann V Propriorezeptives training bei distorsionen des OSG als beitrag zur sekundarprohylaxe und fr üheren wiedereingliederung. [Proprioreceptive training in ankle sprains can contribute to secondary prophylaxis and earlier reintegration] Dtsch Z Sportmed 1999 50 89 91

[B69] Rothermel S Hale SA Hertel J Denegar CR Effect of active foot positioning on the outcome of balance training program Phys Ther Sport 2004 5 98 103

[B70] Brown C Ross S Mynark R Guskiewicz K Assessing functional ankle instability with joint position sense, time to stabilization, and electromyography J Sport Rehabil 2004 13 122 134

[B71] Ross SE Guskiewicz KM Effect of coordination training with and without stochastic resonance stimulation on dynamic postural stability of subjects with functional ankle instability and subjects with stable ankles Clin J Sport Med Offic J Can Acad Sport Med 2006 16 323 328 10.1097/00042752-200607000-00007 16858216

[B72] Wikstrom EA Tillman MD Chmielewski TL Cauraugh JH Naugle KE Borsa PA Dynamic postural control but not mechanical stability differs among those with and without chronic ankle instability Scand J Med Sci Sports 2010 20 e137 e144 19422654 10.1111/j.1600-0838.2009.00929.x

[B73] Santos MJ Liu W Possible factors related to functional ankle instability J Orthop Sports Phys Ther 2008 38 150 157 18383650 10.2519/jospt.2008.2524

[B74] Michell TB Ross SE Blackburn JT Hirth CJ Guskiewicz KM Functional balance training, with or without exercise sandals, for subjects with stable or unstable ankles J Athl Train 2006 41 393 398 17273464 PMC1748421

[B75] Mckeon PO Hertel J Spatiotemporal postural control deficits are present in those with chronic ankle instability BMC Musculoskelet Disord 2008 9 76 18518994 10.1186/1471-2474-9-76 PMC2438356

[B76] Mitchell A Dyson R Hale T Abraham C Biomechanics of ankle instability. Part 2: postural sway-reaction time relationship Med Sci Sports Exerc 2008 40 1522 1528 18614937 10.1249/MSS.0b013e31817356d6

