# Supplementary material for: Study protocol: the effect of whole body vibration on acute unilateral unstable lateral ankle sprain- a biphasic randomized controlled trial
Source: BMC Musculoskelet Disord. 2013 Jan 14;14:22. doi: 10.1186/1471-2474-14-22 (PMC3567953; doi:10.1186/1471-2474-14-22)
Supplement: Additional file 1 — Detailed WBV treatment protocol. [file 1471-2474-14-22-S1.pdf]

Galileo® Training

RCT on WBV and Acute Lateral Ankle Sprain

Trainingsumfang

Siehe Studienprotokoll.

Trainingsparameter

Die **Trainingszeit** wird am Gerät eingestellt. Wählen Sie aus den unten beschriebenen Übungen drei pro Übungsreihe aus. Führen Sie jede Übung 1 Minute durch.

Die **Amplitude**, also die Auslenkung der Trainingsplattform nach oben oder unten, wird über die Fußposition gewählt. Je weiter die Fußposition, desto anspruchsvoller das Training.

Die **Trainingsfrequenz** wird am Gerät eingestellt und wird bestimmt durch das Trainingsziel (Mobilisation, Training der Muskelfunktion oder Steigerung der Muskelleistung).

Hinweise

- Vor dem ersten Training:
  Ausschluss von Gegenanzeigen und Einweisung durch einen geschulten Betreuer.
- Kopf soll nicht mitvibrieren!
  Abhilfe: Engere Fußposition wählen oder Knie beugen!
- Keine durchgestreckten Knie bei Fußpositionen über Position 2,5.
- Fußposition immer symmetrisch um Position 0 einnehmen.
- Alle Übungen langsam und bewusst durchführen.

Tag 4 bis 10

Übung 1
5:00 | 10/16 Hz | 1–2

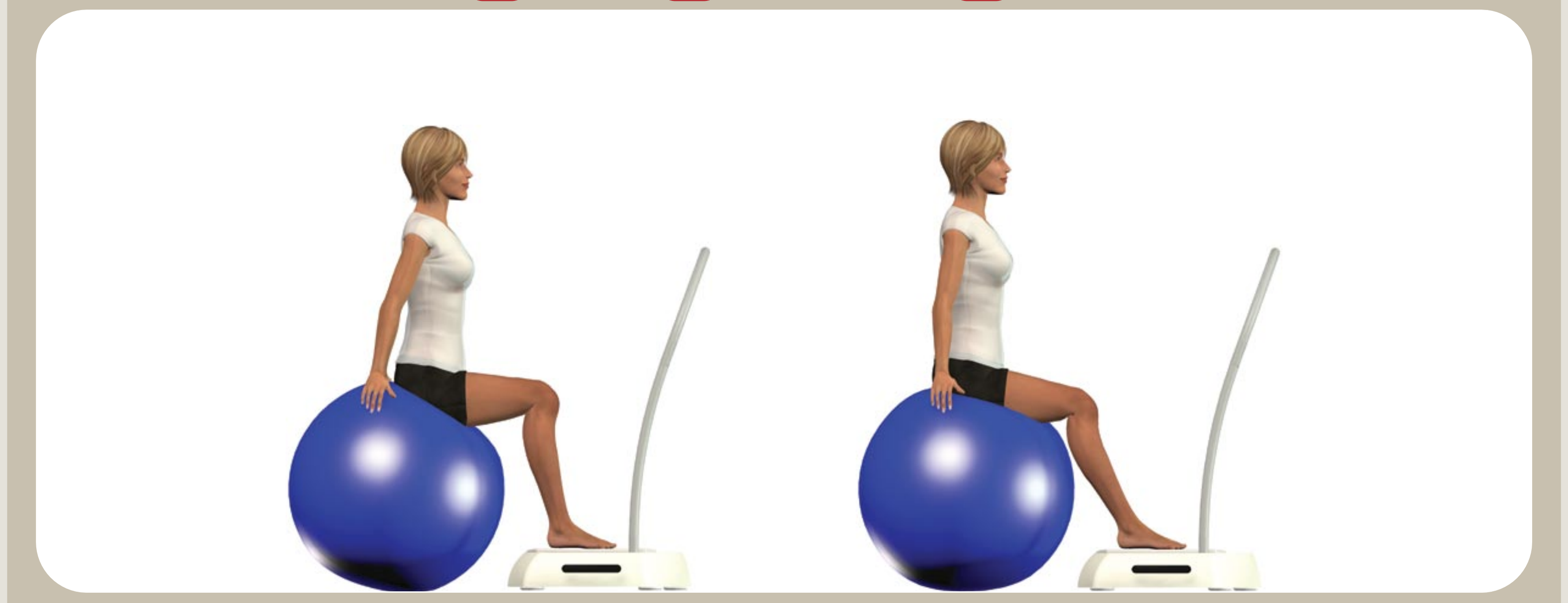

31 Sitz auf Gymnastikball

Aufrechter Sitz.
Ball durch Beugen und Strecken der Knie vor- und zurückrollen.
Oberkörper bleibt aufrecht.
4 Sekunden vorwärts – 4 Sekunden rückwärts.

Übung 2
5:00 | 10/16 Hz | 1–2

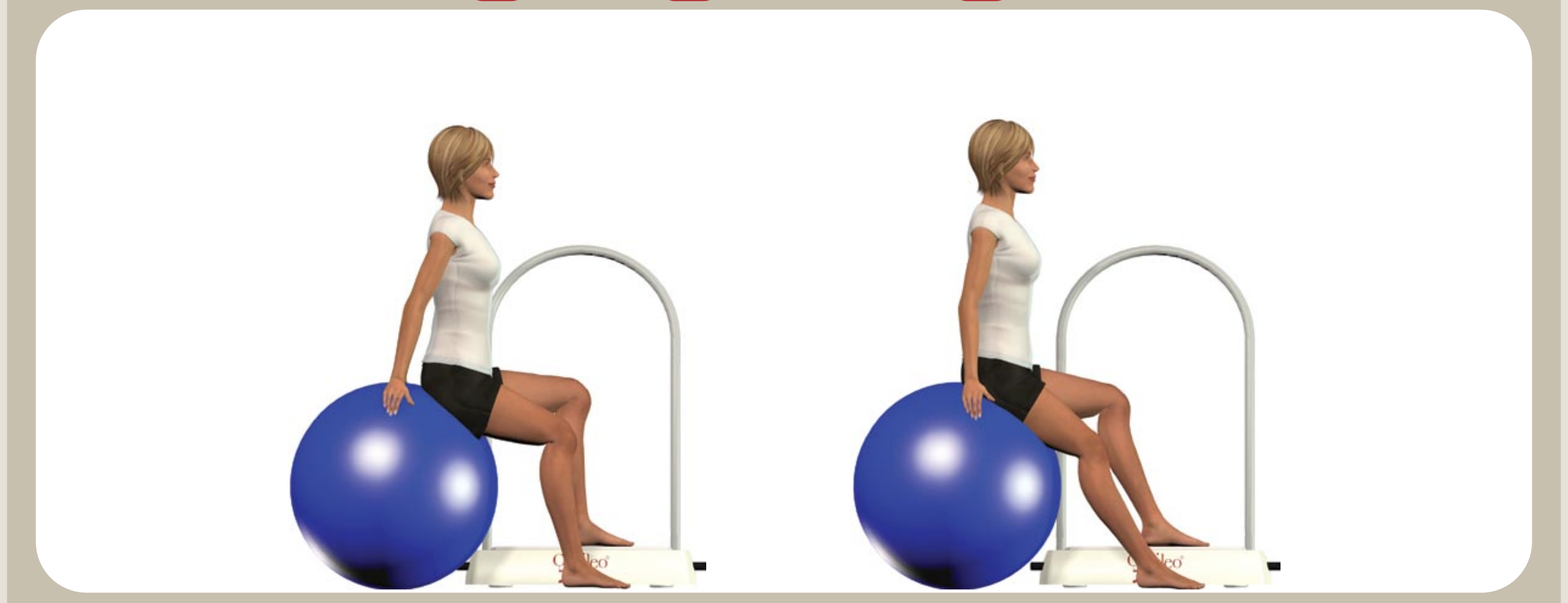

9 Sitz auf Gymnastikball einbeinig

Aufrechter Sitz quer vor Galileo.
Ein Fuß auf die Trainingsplattform, Sprunggelenk auf Position 0!
Ball durch Beugen und Strecken der Knie vor- und zurückrollen.
Oberkörper bleibt aufrecht.
4 Sekunden vorwärts – 4 Sekunden rückwärts.

Tag 14 bis 21

Übung 1
3:00 | 18–24 Hz | 1–2,5

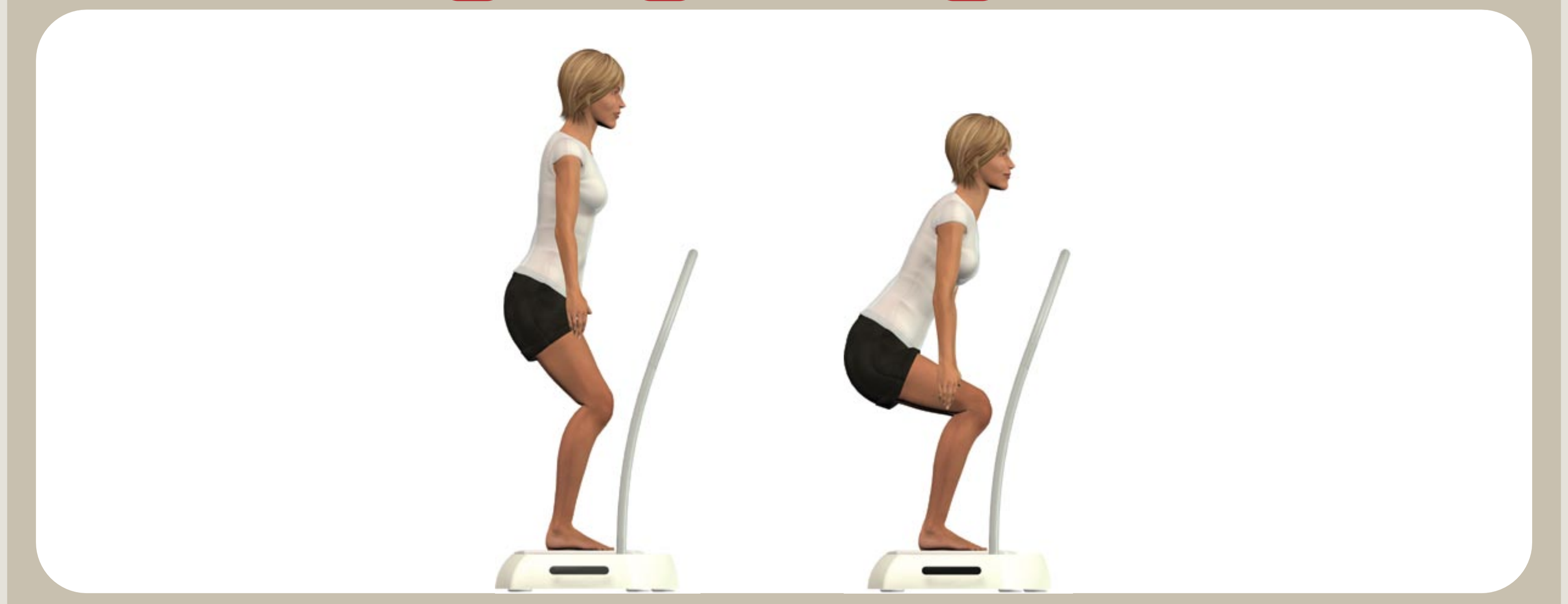

B1 Kniebeuge beidbeinig

Oberkörper aufrecht.
Arme hängen entspannt.
In die Hocke gehen – Fersen bleiben unten!
4 Sekunden abwärts – 4 Sekunden aufwärts.
Auf Beinachse achten!

Übung 2
3:00 | 18–24 Hz | 1–2,5

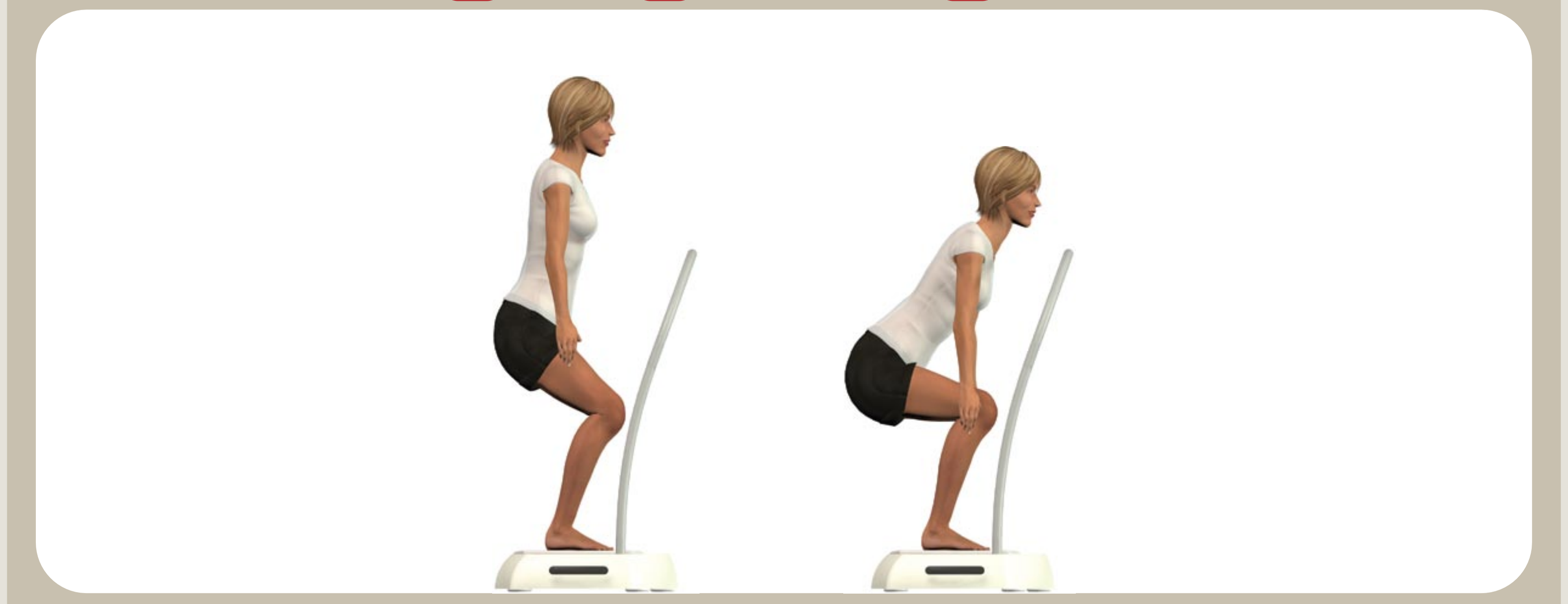

23 Kniebeuge beidbeinig tief

Tiefe Hockposition einnehmen.
Fersen bleiben unten!
Um diese Position 10 cm aufwärts und abwärts bewegen.
4 Sekunden abwärts – 4 Sekunden aufwärts.
Auf Beinachse achten!

Übung 3
3:00 | 18–24 Hz | 1–2,5

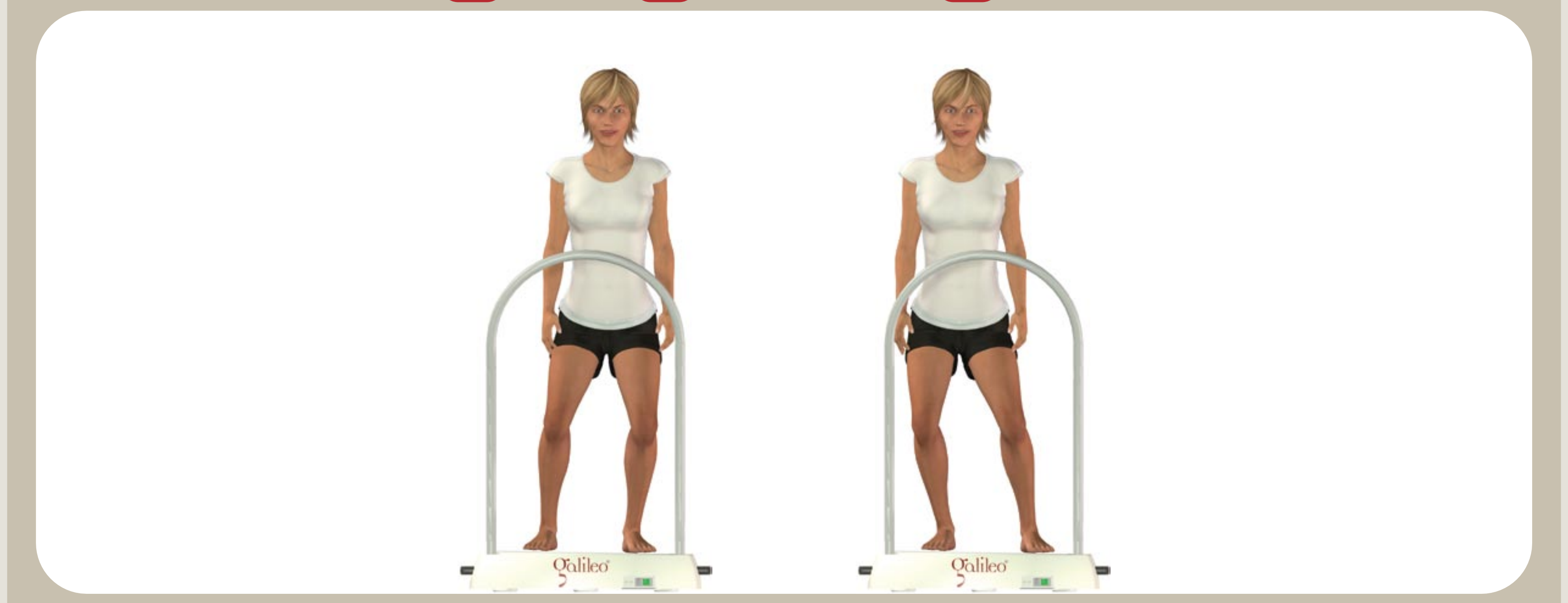

111 Gewichtsverlagerung

Oberkörper aufrecht.
Körpergewicht zur Seite verlagern.
Im Wechsel je 4 Sekunden pro Seite.
Gegebenenfalls Augen schließen.
Keine Seitneigung im Oberkörper!

Übung 4
3:00 | 18–24 Hz | 1–2,5

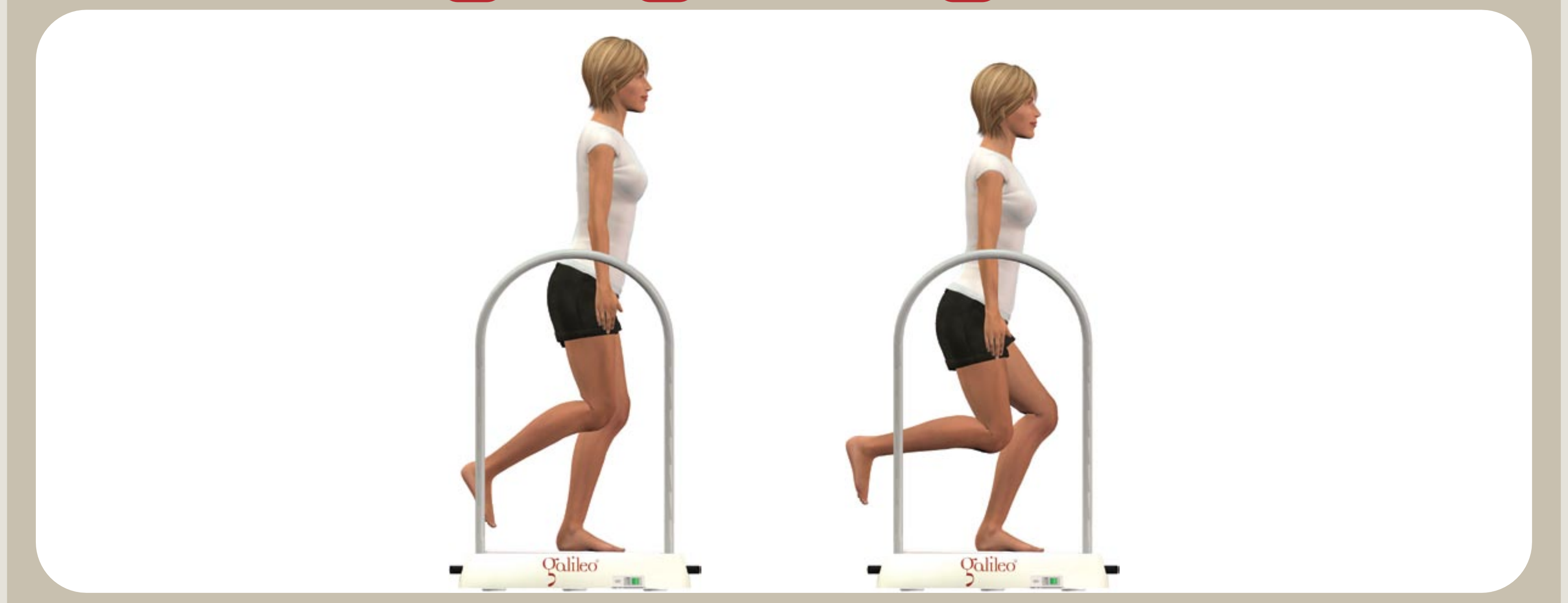

42 Kniebeuge einbeinig quer

Oberkörper aufrecht.
Ein Bein anheben. Sprunggelenk auf Position 0!
Langsame Kniebeuge ausführen. 4 Sek. abwärts – 4 Sek. aufwärts.
Nach der Hälfte der Trainingszeit Bein wechseln.
Auf Beinachse achten!

Insg. 3 Übungsreihen: 2 Minuten Pause nach jeder Übungsreihe, dann Übung 1–4 wiederholen!

© 2012 Novotec Medical GmbH • DocID: 631N00XXDE00A_1

Galileo finden Sie im Internet unter www.galileo-training.com
Novotec Medical GmbH • Durlacher Str. 35 • 75172 Pforzheim • Tel. 07231 15448-30 • Fax 07231 15448-48 • E-Mail info@novotecmedical.de

NOVOTEC MEDICAL – Get fit. Stay fit.

Galileo® Training

RCT on WBV and Acute Lateral Ankle Sprain

Trainingsumfang

Siehe Studienprotokoll.

Trainingsparameter

Die **Trainingszeit** wird am Gerät eingestellt. Wählen Sie aus den unten beschriebenen Übungen drei pro Übungsreihe aus. Führen Sie jede Übung 1 Minute durch.

Die **Amplitude**, also die Auslenkung der Trainingsplattform nach oben oder unten, wird über die Fußposition gewählt. Je weiter die Fußposition, desto anspruchsvoller das Training.

Die **Trainingsfrequenz** wird am Gerät eingestellt und wird bestimmt durch das Trainingsziel (Mobilisation, Training der Muskelfunktion oder Steigerung der Muskelleistung).

Hinweise

- Vor dem ersten Training:
  Ausschluss von Gegenanzeigen und Einweisung durch einen geschulten Betreuer.
- Kopf soll nicht mitvibrieren!
  Abhilfe: Engere Fußposition wählen oder Knie beugen!
- Keine durchgestreckten Knie bei Fußpositionen über Position 2,5.
- Fußposition immer symmetrisch um Position 0 einnehmen.
- Alle Übungen langsam und bewusst durchführen.

Woche 4 bis 6

Übung 1

3:00 | 18–24 Hz | 2–3

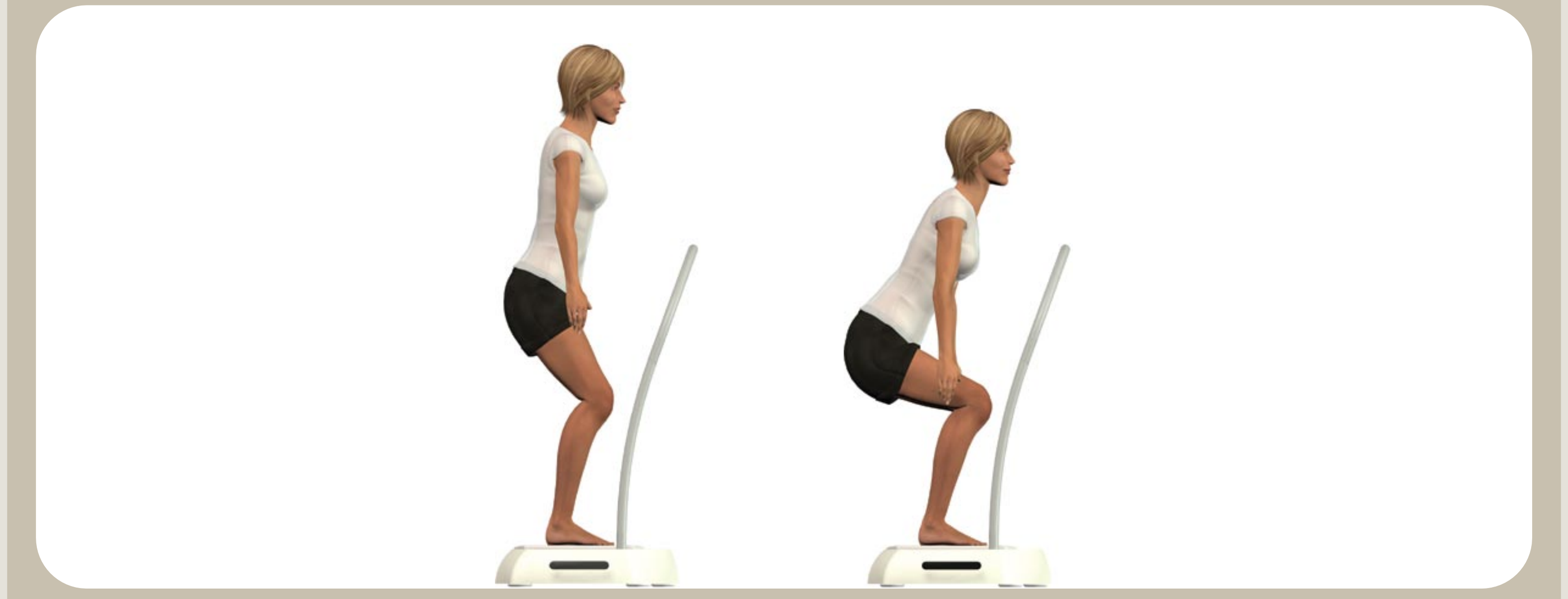

B1 Kniebeuge beidbeinig

Oberkörper aufrecht.
Arme hängen entspannt.
In die Hocke gehen – Fersen bleiben unten!
4 Sekunden abwärts – 4 Sekunden aufwärts.
Auf Beinachse achten!

Übung 2

3:00 | 18–24 Hz | 2–3

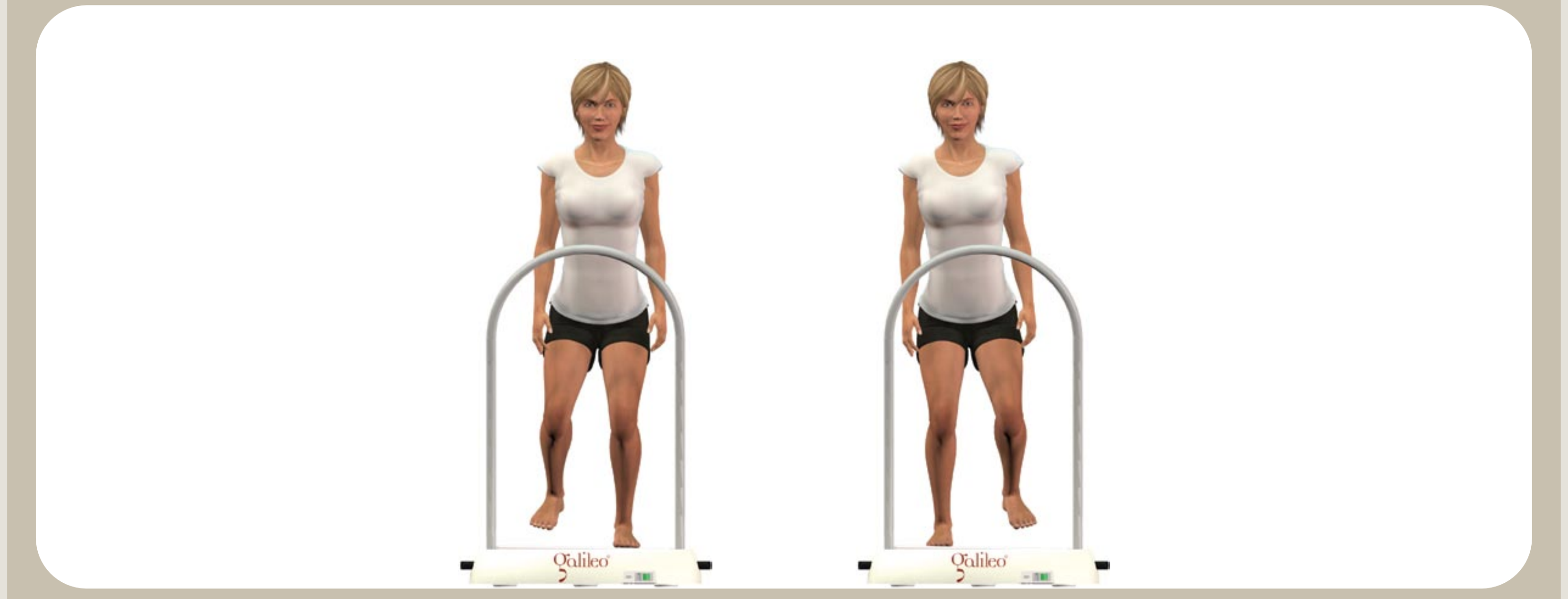

43 Hüpfen im Wechsel

Oberkörper aufrecht.
Im Wechsel auf linkes und rechtes Bein hüpfen.
Keine Seitneigung im Oberkörper!

Übung 3

3:00 | 18–24 Hz | 2–3

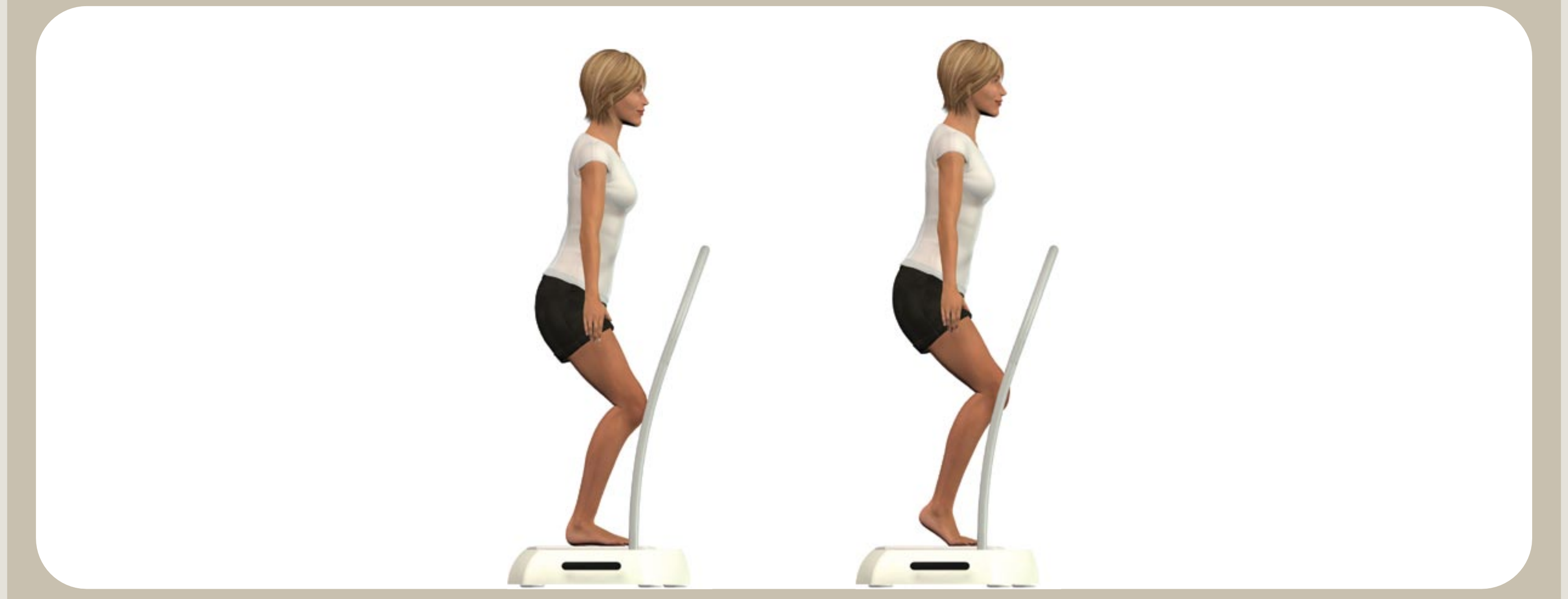

1 Wadentraining Kniebeuge beidbeinig

Oberkörper aufrecht. Knie stark beugen.
Fersen so weit wie möglich anheben.
Endposition 20 Sekunden halten.
Fersen langsam in Ausgangsposition zurück.
Übung wiederholen.

Übung 4

3:00 | 18–24 Hz | 2–3

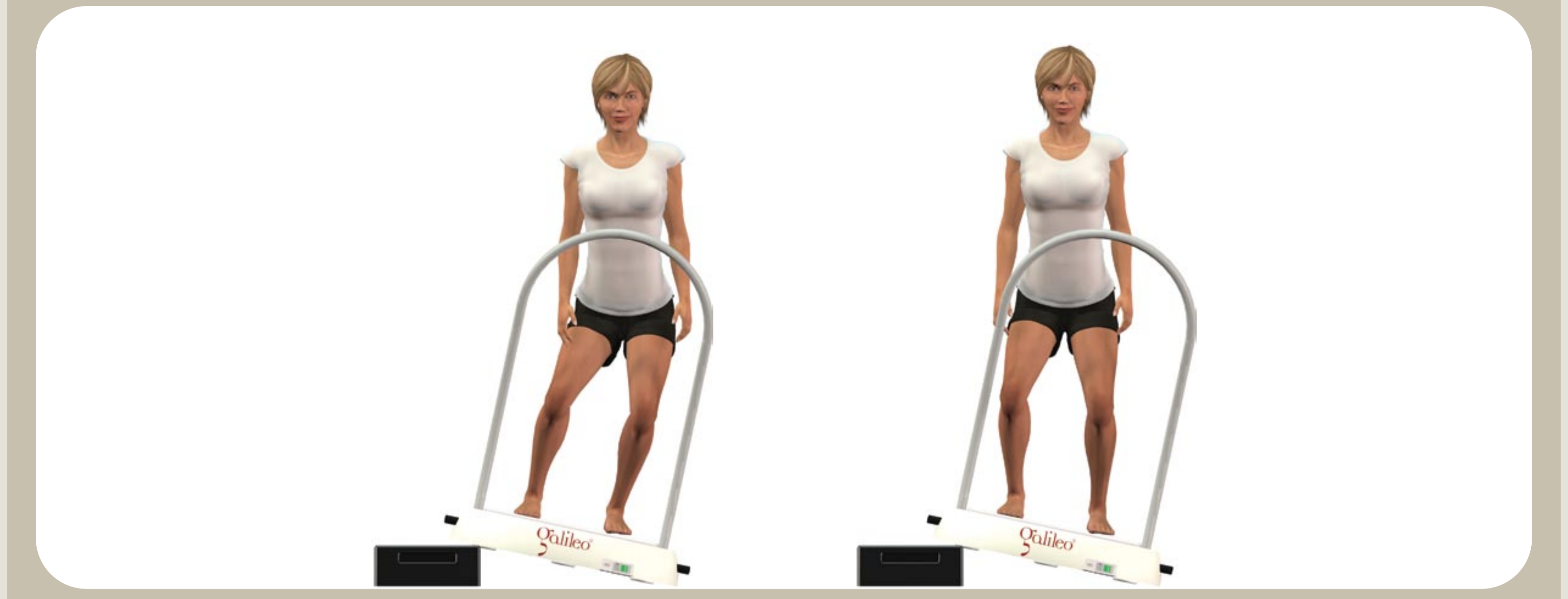

10 Gewichtsverlagerung einseitig unterlegt

Galileo einseitig unterlagern.
Oberkörper aufrecht.
Körpergewicht zur Seite verlagern.
Im Wechsel je 4 Sekunden pro Seite.
Keine Seitneigung im Oberkörper!

Übung 5

3:00 | 18–24 Hz | 2–3

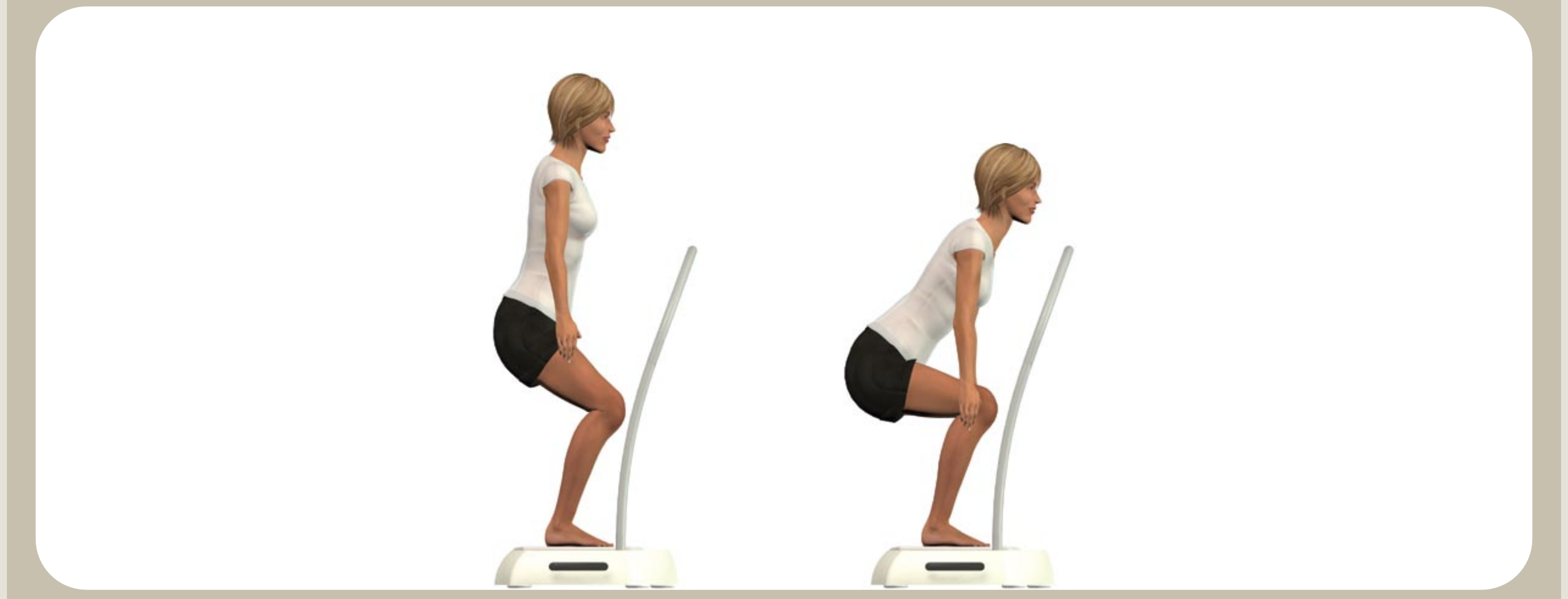

23 Kniebeuge beidbeinig tief

Tiefe Hockposition einnehmen (45°–90°).
Fersen bleiben unten!
Tiefe Hocke solange wie möglich halten.
Auf Beinachse achten!

Insg. 3 Übungsreihen: 2 Minuten Pause nach jeder Übungsreihe, dann Übung 1–5 wiederholen!

© 2012 Novotec Medical GmbH • DocID: 631N00XXDE00A_2

Galileo finden Sie im Internet unter www.galileo-training.com
Novotec Medical GmbH • Durlacher Str. 35 • 75172 Pforzheim • Tel. 07231 15448-30 • Fax 07231 15448-48 • E-Mail info@novotecmedical.de

NOVOTEC MEDICAL – Get fit. Stay fit.
